# Role of Cardio-Renal Dysfunction, Inflammation Markers, and Frailty on In-Hospital Mortality in Older COVID-19 Patients: A Cluster Analysis

**DOI:** 10.3390/biomedicines11092473

**Published:** 2023-09-06

**Authors:** Francesco Spannella, Federico Giulietti, Giorgia Laureti, Mirko Di Rosa, Chiara Di Pentima, Massimiliano Allevi, Caterina Garbuglia, Piero Giordano, Matteo Landolfo, Letizia Ferrara, Alessia Fumagalli, Fabrizia Lattanzio, Anna Rita Bonfigli, Riccardo Sarzani

**Affiliations:** 1 Internal Medicine and Geriatrics, IRCCS INRCA, 60127 Ancona, Italy; 2 Department of Clinical and Molecular Sciences, “Politecnica delle Marche” University, 60126 Ancona, Italy; 3 Geriatric Pharmacoepidemiology and Biostatistics, IRCCS INRCA, 60127 Ancona, Italy; 4 Medical Direction, Risk Manager, IRCCS INRCA, 60127 Ancona, Italy; 5 Pulmonary Rehabilitation Unit, IRCCS INRCA, 23880 Casatenovo, Italy; 6 Scientific Direction, IRCCS INRCA, 60127 Ancona, Italy

**Keywords:** COVID-19, SARS-CoV-2, cluster, NT-proBNP, older adults, in-hospital mortality

## Abstract

Our study aimed to identify clusters of hospitalized older COVID-19 patients according to their main comorbidities and routine laboratory parameters to evaluate their association with in-hospital mortality. We performed an observational study on 485 hospitalized older COVID-19 adults (aged 80+ years). Patients were aggregated in clusters by a K-medians cluster analysis. The primary outcome was in-hospital mortality. Medical history and laboratory parameters were collected on admission. Frailty, defined by the Clinical Frailty Scale (CFS), referred to the two weeks before hospitalization and was used as a covariate. The median age was 87 (83–91) years, with a female prevalence (59.2%). Three different clusters were identified: cluster 1 (337), cluster 2 (118), and cluster 3 (30). In-hospital mortality was 28.5%, increasing from cluster 1 to cluster 3: cluster 1 = 21.1%, cluster 2 = 40.7%, and cluster 3 = 63.3% (*p* < 0.001). The risk for in-hospital mortality was higher in clusters 2 [HR 1.96 (95% CI: 1.28–3.01)] and 3 [HR 2.87 (95% CI: 1.62–5.07)] compared to cluster 1, even after adjusting for age, sex, and frailty. Patients in cluster 3 were older and had a higher prevalence of atrial fibrillation, higher admission NT-proBNP and C-reactive protein levels, higher prevalence of concurrent bacterial infections, and lower estimated glomerular filtration rates. The addition of CFS significantly improved the predictive ability of the clusters for in-hospital mortality. Our cluster analysis on older COVID-19 patients provides a characterization of those subjects at higher risk for in-hospital mortality, highlighting the role played by cardio-renal impairment, higher inflammation markers, and frailty, often simultaneously present in the same patient.

## 1. Introduction

Age and multimorbidity are major risk factors for death in patients affected by coronavirus disease 2019 (COVID-19) [1,2]. Since the beginning of the pandemic, older patients have exhibited susceptibility to developing more aggressive disease courses, with a higher risk of death [3]. It is essential to establish objective criteria to stratify older COVID-19 patients at higher risk of death to improve patient management. In addition to older age, several studies have shown that comorbidities, especially cardiovascular (CV) diseases such as arterial hypertension and diabetes mellitus, are among the most relevant negative prognostic factors for COVID-19 [4]. At the same time, patients with heart failure (HF) and chronic kidney disease (CKD), highly prevalent conditions in older subjects [5], are particularly susceptible to COVID-19 complications [6,7]. Patients aged 75 years and older have high rates of in-hospital complications (respiratory failure, HF, renal failure, and sepsis) that are significantly associated with higher mortality [8]. In previous cohorts of geriatric COVID-19 patients, routine laboratory biomarkers on admission were also associated with in-hospital mortality. The proportion of patients with the highest neutrophil counts, both absolute and relative, was significantly higher among the deceased patients, whereas the opposite trend was observed for lymphocytes [9]. Furthermore, age-related changes and frailty play an important role in the prognosis of the disease [10,11]. Therefore, many risk factors for mortality have been identified in older COVID-19 patients; however, it has not yet been clarified whether and which of these factors can converge in the same patient, determining a worse prognosis.

In this observational study, we aimed to identify clusters of older COVID-19 patients according to their main comorbidities and clinical and laboratory parameters in order to evaluate their association with in-hospital mortality.

## 2. Materials and Methods

### 2.1. Study Design and Population

For the present study, we extrapolated data from the Report-Age COVID project, which is an observational register of older adults consecutively admitted from March 2020 to March 2021 into the COVID-19 wards of the Italian National Institute of Health and Science on Ageing (INRCA: Istituto Nazionale di Riposo e Cura per Anziani); the only organization specifically focused on geriatric care and gerontological research in Italy. Our hospital is dedicated to scientific research and the care of older subjects (mostly aged 80 years and older); therefore, we advocate for studies on older patients affected by COVID-19, aiming to increase the level of scientific evidence, which is lower in this age group than younger populations as older subjects are still often excluded from clinical trials. We applied the following inclusion criteria: age over 80 years, access from the emergency department with a confirmed SARS-CoV-2 infection following a standard molecular test using reverse transcriptase-polymerase chain reaction (RT-PCR) from nasal/oro-pharyngeal swab on admission, and availability of laboratory and clinical data of interest. No particular exclusion criteria were applied in this study.

### 2.2. Ethics Statement

Clinical investigations have been conducted according to the principles expressed in the Declaration of Helsinki and its later amendments. The Report-Age protocol study has been approved by the Ethics Committee of our institution (CE INRCA, reference number CE-INRCA-20008) and registered under the ClinicalTrials.gov database (reference number NCT04348396). All statistical analyses were performed with anonymized data. All research was performed in accordance with the relevant guidelines and regulations.

### 2.3. Clinical Parameters

Medical history and laboratory parameters were collected from each enrolled patient on admission. We took into account the following comorbidities: diabetes mellitus, arterial hypertension, dyslipidemia, chronic lung diseases, atrial fibrillation, myocardial infarction, dementia, and stroke. Dyslipidemia, hypertension, chronic lung diseases, and diabetes mellitus were defined according to the individual medical history and/or the respective treatments taken at home. Systolic blood pressure (BP) and the presence of a concurrent bacterial infection on admission were also taken into account. Concurrent bacterial infections were diagnosed by the attending physician based on a combination of symptoms, clinical examination, imaging tests, and bacteriological tests. The following laboratory admission parameters found to be associated with organ damage and COVID-19 severity in previous studies were also taken into account: creatinine and glomerular filtration rate (GFR, estimated using the CKD-EPI equation) as markers of renal dysfunction, N-terminal pro-B-type natriuretic peptide (NT-proBNP) as a marker of cardiac dysfunction, and neutrophil count and C-reactive protein (CRP) as markers of infection/inflammation. The Charlson comorbidity index was calculated as previously described [12]. Frailty was graded according to the Clinical Frailty Scale (CFS), evaluating functional abilities two weeks before hospital admission [13]. The CFS is an ordinal scale that ranks frailty from 1 to 9 (from being very fit to terminally ill), with higher scores indicating progressively higher degrees of frailty.

### 2.4. Statistical Analysis

In our study population, cluster analysis was chosen to explore the subjects’ characteristic combinations; in fact, this method allowed us to group individuals in different clusters and better capture the complexity of hospitalized older COVID-19 patients. Patients were aggregated in clusters by K-medians cluster analysis. The identification of the optimal number of clusters ranged between 2 and 5 clusters and was carried out using the Calinski-Harabasz cluster validity index. The best solution was 3 clusters (Calinski-Harabasz pseudo-F maximum value = 799.21, other values ranged between 584.09 and 797.98). Continuous variables were reported as either mean and standard deviation or median and interquartile range based on their distribution (assessed using the Shapiro-Wilk test). The comparison of variables among clusters was performed via one-way analysis of variance (ANOVA) or Kruskal-Wallis equality-of-populations rank test according to their distribution. The categorical variables were expressed as an absolute number and percentage and analyzed by Chi-square test. The association between clusters and in-hospital mortality was explored by Kaplan-Meier survival curves, and the statistical significance was assessed using a Log-rank test for equality of survivor functions. The association of each independent variable and cluster with in-hospital mortality was analyzed by Cox proportional hazard models adjusted for age, sex, and frailty. The accuracy of clusters and frailty in predicting mortality was estimated by the area under the Receiver Operating Characteristic (ROC) curve. Finally, the additive effect of the CFS on the predictive ability of the clusters was investigated. Changes in the area under the curve (AUC) and categorical net reclassification index (NRI) with 1000 bootstrap samples were calculated to estimate 95% CIs. Statistical analysis was conducted using the Stata 15.1 Software Package for Windows (StataCorp, College Station, TX, USA).

## 3. Results

### 3.1. General Characteristics and Cluster Characterization

The general characteristics of the study population are described in Table 1. A total of 485 patients aged 80+ years were included in the study. The median age was 87 (83–91) years, with a female prevalence (59.2%). The median length of stay was 14 (9–22) days. Hypertension was the most prevalent comorbidity, followed by dementia and dyslipidemia. The following factors were applied in the cluster analysis: age, sex, diabetes mellitus, hypertension, atrial fibrillation, dementia, myocardial infarction, concurrent bacterial infection, stroke, dyslipidemia, NT-proBNP, creatinine, neutrophils, eGFR, CRP, and systolic BP. Three different clusters were identified in our population of hospitalized older COVID-19 patients: cluster 1 (337 patients), cluster 2 (118 patients), and cluster 3 (30 patients). Cluster 1 was the most numerous and characterized by both a lower median age and CFS compared to the other clusters. The patients in cluster 1 had lower levels of both NT-proBNP and CRP and a higher eGFR. Moving from cluster 1 to cluster 3, we found an increase in the prevalence of atrial fibrillation and concurrent bacterial infection, as well as an increase in NT-proBNP and CRP levels, together with a decrease in eGFR levels.

### 3.2. In-Hospital Mortality

**Figure 1:**
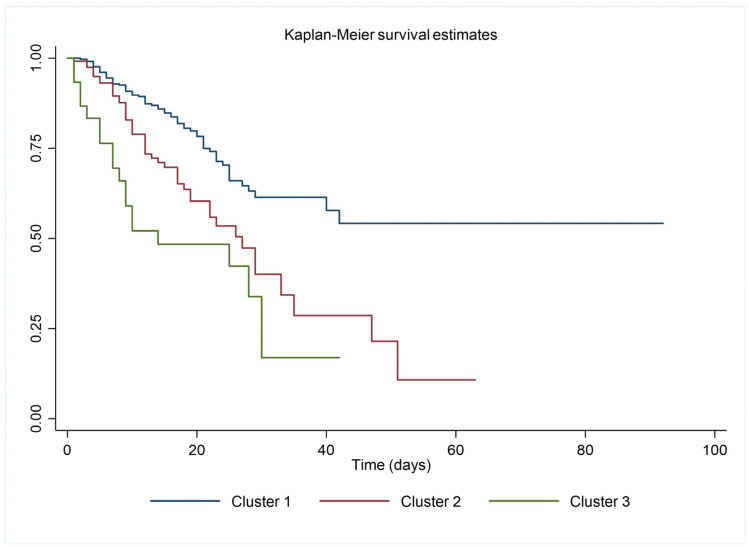
Log-rank test for equality of survivor functions: χ^2^ = 30.01; *p* < 0.001.

**Figure 2:**
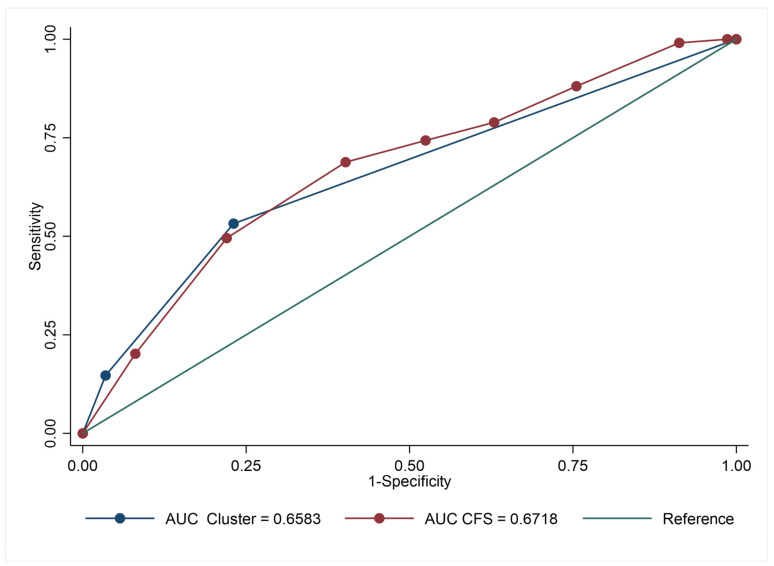
Accuracy comparison for Cluster and CFS.

Death occurred in 28.5% of the study population. In-hospital mortality increased from cluster 1 to cluster 3 (Table 1). The Kaplan-Meier survival curves describe the association between the clusters and in-hospital mortality (Figure 1). Cluster 3 had the highest risk of in-hospital mortality, followed by cluster 2 and cluster 1 (Table 2). Among the comorbidities and the laboratory parameters, a higher NT-proBNP, higher neutrophil count, CRP, and a lower eGFR were associated with a higher risk of in-hospital mortality, even after adjusting for age, sex, and frailty (Table 2). After analyzing the accuracy of predicting mortality in ROC curves, clusters and CFS showed similar AUCs (Figure 2). The addition of CFS led to a significant improvement of the AUC and NRI on the predictive ability of the clusters for in-hospital mortality, as described in Table 3.

## 4. Discussion

Several considerations can be drawn from our cluster analysis of a wide, older population aged 80+ years, hospitalized for COVID-19: (i) older COVID-19 patients can be clustered in three groups according to the main routine clinical and laboratory parameters, (ii) cardiac and renal impairment, together with high inflammation indices, are often present simultaneously in the same patient and are closely associated with in-hospital mortality, and (iii) the assessment of frailty adds predictive power in this context.

Despite a large number of studies that have evaluated prognostic risk factors [14,15], only a few studies have been performed thus far that clustered older patients hospitalized for COVID-19. Mahmoud M. et al. performed a study to identify comorbidity clusters associated with in-hospital mortality in a cohort of older COVID-19 patients (mean age: 83 ± 6.2 years) who were admitted to hospital during the first pandemic wave, although without taking into account any laboratory parameters [16]. They identified three clusters: an unspecified cluster, a metabolic-renal-cancer cluster with higher proportions of hypertension, diabetes, kidney disease, previous active cancer, and chronic obstructive pulmonary disease, and a neurocognitive cluster with higher proportions of heart disease, dementia, and cerebral-vascular disease or vascular diseases. These last two clusters showed a higher mortality rate. Clustering allows for maximization of the distance between groups of patients and grouping of their characteristics while increasing the similarity within each group. The older the population, the more difficult the classification into “pure” clusters according to each disease (i.e., cardiovascular, neurological, pulmonary, renal, endocrine, and metabolic diseases), although theoretically interesting, given the high prevalence of multimorbidity in older patients where multiple strongly interconnected organs and systems are affected at the same time [17,18]. Moreover, this is out of our purpose, and it is far from the “geriatric vision”, which focuses on the whole patient and not on a single disease.

In our study, after also taking into account routine laboratory parameters, it emerged that NT-proBNP, CRP, and eGFR are likely to cluster in an older population and affect mortality. These are typical markers of disease severity and cardio-renal impairment in older patients affected by COVID-19. Our findings are in line with previous reports. In a study on 113 deceased COVID-19 patients, common complications included acute cardiac injury (77%), HF (49%), acute kidney injury (25%), and hypoxic encephalopathy (20%), and patients with CV comorbidity were more likely to develop cardiac complications [19]. A previous meta-analysis that summarized studies on younger populations concluded that these biomarkers of inflammation and cardiac and renal impairment permit the identification of different phenotypes in patients and may predict COVID-19 prognosis [20]. Both high troponin and NT-proBNP levels are associated with COVID-19-related cardiac complications and a poor prognosis, regardless of the previous known history of HF [21,22,23]. In a large cohort of COVID-19 patients, 48.5% had NT-proBNP levels above the recommended cut-off values for the identification of acute HF as recommended by the Heart Failure Association of the European Society of Cardiology, and NT-proBNP was independently associated with all-cause mortality at a median follow-up of 53 days after adjusting for all potentially relevant confounders [HR 1.28 (1.13–1.44) per logarithmic unit] [24]. 

As well as abnormal cardiac biomarkers, left-ventricular and right-ventricular dysfunction, assessed by transthoracic echocardiography on admission, are also associated with a poor prognosis in COVID-19 patients [25]. If this is true for an adult, it is even more so for an older subject. In our previous study on a similar older population (mean age: 88.1 ± 5.1 years) hospitalized for several acute medical illnesses, including bacterial pneumonia and sepsis, we found that higher levels of NT-proBNP on admission were associated with the risk of in-hospital mortality. Indeed, patients with an NT-proBNP ≥ 1800 pg/mL had more than a 2-fold higher risk of in-hospital mortality than their counterparts [5]. This reflects that the increase of this cardiac biomarker and its prognostic power are independent of COVID-19 but common in older subjects in general. Multiple pathophysiological mechanisms are responsible for the elevation of NT-proBNP levels during COVID-19. Although the high NT-proBNP levels in an older population could be, at least in part, due to other comorbidities, they still reflect the presence of cardiac overload/stress. Whenever an acute intercurrent illness increases the body’s metabolic demands, the development of underlying HF is highly probable and associated with a worsened outcome, especially in older, comorbid patients with a limited cardiac reserve [26]. 

Moreover, pneumonia may lead to right ventricular strain and higher intracavitary pressures in the right cardiac chambers. Despite early evidence of an infiltration of interstitial macrophages in the myocardium, only a minority of patients have a direct myocardial injury according to the current criteria for myocarditis [27]. More broadly, natriuretic peptides, including NT-proBNP, are global and most often used for the diagnosis, stratification, and prognosis of HF in both acute and chronic settings, regardless of SARS-CoV-2 infection. They should be measured in all patients who present with symptoms suggestive of new-onset or worsening HF, such as dyspnea and/or fatigue because their use facilitates both the early diagnosis and the early exclusion of HF. These biomarkers can easily support the exclusion of HF due to their very high negative predictive value (94–97%) [28].

The age-related structural and functional changes of the kidney impair its ability to withstand and recover from injury, thus contributing to the high prevalence of CKD and the high susceptibility of older subjects to acute kidney injury (AKI). According to a meta-analysis of 38 studies with 42,779 patients, an older age was a significant risk factor for AKI in COVID-19 [29]. Another meta-analysis of 42 studies enrolling 8932 participants evaluated the association of CKD and AKI with the clinical prognosis of COVID-19 patients. It found that patients with CKD had a significantly higher risk of progression to severe disease (OR 2.31, 95% CI 1.64–3.24) or death (OR 5.11, 95% CI 3.36–7.77) compared to patients without previously diagnosed CKD. Moreover, age had a significant impact on the association between CKD and disease severity [30]. Our results are in line with these previous studies, where impaired renal function had a negative prognostic significance. According to a retrospective analysis of 555 COVID-19 patients, kidney injury, defined by the presence of proteinuria, hematuria, and AKI, was common in COVID-19 and associated with increased mortality [31]. Kidney damage in COVID-19 is multifactorial, involving direct viral infection, indirect injury by sepsis, hemodynamic and cardiac alterations, cytokine storm, disseminated intravascular coagulation, and other unknown mechanisms. Moreover, histopathologic analyses have suggested the possibility that SARS-CoV-2 can directly infect the renal tubular epithelium [32,33].

Moreover, an elevated CRP, alone or in addition to other inflammatory biomarkers, has been proposed as a predictor of COVID-19 severity in previous studies, in agreement with our data [34]. In a meta-analysis of 56 studies involving 8719 patients with confirmed COVID-19, CRP was significantly higher in severe disease and in patients who died during follow-up [35]. Therefore, monitoring inflammatory markers may serve as an early warning system for progression to severe COVID-19. In a recent study on a similar older population, the blood count parameters and some circulating biomarkers of inflammation had the best performance in predicting short-term mortality [9]. At the same time, CRP levels, together with pro-calcitonin levels, can help to detect superimposed bacterial infections earlier, improving the appropriateness of antibiotic therapy. A large retrospective study in the ICU on 17,534 COVID-19 patients found a mortality rate almost five times higher in patients with secondary infections compared to patients with no secondary infections [36]. Similarly, the prevalence of concurrent bacterial infections tended to be higher in the cluster with the highest mortality in our study (cluster 3). Bacterial infections could also prolong hospitalization and complicate the clinical course.

Our study also reinforces the role of frailty in affecting COVID-19 prognosis. Indeed, the addition of CFS led to a significant improvement of the AUC and NRI on the predictive ability of the clusters for in-hospital mortality. Frailty predicts mortality in both adult and older populations hospitalized for acute respiratory illness [37]. The complexity and pathogenicity of COVID-19 in advanced age go through impaired homeostasis, biomarkers of frailty, and diminished organ reserve, especially for the cardiovascular system. Our older patients were the prototypes of frail and comorbid subjects, in which impaired cardiac and/or renal function, along with the augmented risk of concurrent bacterial infections, contributed to the worst outcomes. An Italian retrospective study on 165 patients admitted to an acute geriatric ward found that frailty, assessed with CFS, was able to identify those COVID-19 patients at risk for in-hospital death regardless of age. Multimorbidity, impaired immune defense, sarcopenia, and malnutrition are likely to play key roles in this process [38].

### Study Limitations

To the best of our knowledge, only a few previous studies used cluster analysis to investigate older patients hospitalized for COVID-19. The main strength of our study is the older age of the study population, for which the level of scientific evidence is low. Moreover, we took into account both the most common comorbidities and laboratory parameters in our analysis, allowing a wide applicability of our findings in routine clinical practice. However, several limitations need to be addressed. The main limitation is the observational nature of the study, which does not allow us to evaluate the causal effect of the relations found. 

Moreover, we took into account only the laboratory parameters measured on admission, as data regarding the trend of these parameters during hospitalization were not available. The variable “obesity”, a known risk factor for severe COVID-19 [1], was not available in our registry and, therefore, not taken into account in the analyses. Indeed, the accurate measurement of both weight and height was not systematically feasible during the data collection of the Report-Age COVID registry in an acute and emergency setting on severely compromised older patients, often bedridden on admission. In the registry data collection, the variable “concurrent bacterial infection” was entered into the checklist as presence/absence. Therefore, it was not possible to trace the type and site of infection specifically. However, the majority of bacterial infections were bacterial pneumonia, a superinfection of viral pneumonia, and urinary tract infections, which are the two most common types of bacterial infections in this population.

## 5. Conclusions

Determinants of COVID-19 severity and mortality are generally attributed to age and multimorbidity. However, older subjects can show a broad heterogeneity in terms of health status and patterns of multimorbidity. Our cluster analysis provides a better characterization of older COVID-19 patients by using widely available blood tests in clinical practice and evaluating frailty. Our study highlights precisely through the cluster analysis how several factors are often present simultaneously in the same subject, characterizing a type of older patient with a higher risk of death during hospitalization for COVID-19. This patient is characterized by high levels of inflammation markers and high rates of concurrent bacterial infections associated with the simultaneous presence of cardio-renal impairment. This approach (cluster analysis) can be informative, taking into account the high heterogeneity of older people and the multiple interactions between different variables. Our evidence also stresses the key prognostic role played by NT-proBNP, eGFR, and CRP in older COVID-19 patients.

## Figures and Tables

**Table 1:** General characteristics of the study population according to clusters.

	Total	Cluster 1	Cluster 2	Cluster 3	*p*
	N = 485	N = 337	N = 118	N = 30	
Female sex	287 (59.2%)	205 (60.8%)	64 (54.2%)	18 (60.0%)	0.453
Age	87 (83–91)	86 (82–90)	89 (84–94)	88 (84–91)	**<0.001**
CFS	6 (4–8)	6 (4–8)	7 (4–8)	7 (6–8)	**0.025**
Comorbidities					
Diabetes Mellitus	100 (20.6%)	63 (18.7%)	33 (28%)	4 (13.3%)	0.060
Hypertension	318 (65.6%)	211 (62.6%)	90 (76.3%)	17 (56.7%)	**0.015**
Atrial Fibrillation	130 (26.8%)	64 (19%)	52 (44.1%)	14 (46.7%)	**<0.001**
Dementia	168 (34.6%)	111 (32.9%)	45 (38.1%)	12 (40%)	0.485
Chronic Lung Diseases	70 (14.4%)	44 (13.1%)	22 (18.6%)	4 (13.3%)	0.326
Myocardial Infarction	7 (1.4%)	2 (0.6%)	4 (3.4%)	1 (3.3%)	0.061
Concurrent Bacterial Infection	25 (5.2%)	13 (3.9%)	8 (6.8%)	4 (13.3%)	0.052
Stroke	33 (6.8%)	21 (6.2%)	9 (7.6%)	3 (10%)	0.676
Dyslipidemia	160 (33%)	114 (33.8%)	35 (29.7%)	11 (36.7%)	0.643
Charlson Index (points)	1 (0–2)	1 (0–1)	1 (0–2)	1 (0–2)	0.032
Lab Parameters					
NT-proBNP (pg/mL)	1541 (569–4174)	830 (376–1627)	5302.5 (4260–8512)	29,480.5 (21,250–40,161)	**<0.001**
Creatinine (mg/dL)	1.03 (0.66–1.45)	1.08 (0.72–1.5)	0.92 (0.58–1.29)	0.91 (0.57–1.2)	**0.008**
eGFR (ml/min/1.73 m^2^)	69 (45–84)	79 (56–86)	47 (30–74)	33.5 (18–49)	**<0.001**
Neutrophil count (n/microl)	6.14 (4.35–9.4)	5.92 (4.28–8.67)	6.665 (4.53–10.61)	6.88 (4.56–11.31)	0.095
CRP (mg/dL)	3.65 (1.31–8.44)	3.06 (1.17–7.54)	4.31 (1.89–10.01)	6.66 (2.19–13.07)	**0.003**
Systolic BP (mmHg)	135.5 ± 21.5	136.9 ± 20.5	134.9 ± 22.7	122.2 ± 24.5	0.203
End-points					
Length of stay (days)	14 (9–22)	14 (9–22)	13.5 (9–21)	10.5 (5–25)	0.437
In-hospital mortality	138 (28.5%)	71 (21.1%)	48 (40.7%)	19 (63.3%)	**<0.001**

Bold indicates significance. Dyslipidemia, hypertension, chronic lung diseases, and diabetes mellitus were defined according to individual medical history and/or respective treatments taken at home. Diabetes mellitus, hypertension, dementia, dyslipidemia, and concurrent bacterial infection are referred to as presence on admission. Atrial fibrillation, myocardial infarction, and stroke are referred to as previous events in the history of the patient. CFS: clinical frailty scale; NT-proBNP: N-terminal pro-B-type natriuretic peptide; eGFR: estimated glomerular filtration rate; CRP: C-reactive protein; BP: blood pressure.

**Table 2:** Association with in-hospital mortality: univariate Cox regression analysis adjusted for age, sex, and frailty.

	HR (95%CI)
Comorbidities	
Diabetes Mellitus	0.86 (0.54–1.37)
Hypertension	0.82 (0.54–1.24)
Atrial Fibrillation	1.34 (0.90–1.99)
Dementia	1.01 (0.68–1.50)
Chronic Lung Diseases	1.43 (0.91–2.23)
Myocardial Infarction	1.49 (0.37–6.08)
Concurrent Bacterial Infection	0.77 (0.37–1.59)
Stroke	0.68 (0.32–1.48)
Dyslipidemia	**0.64 (0.41–0.99)**
Lab Parameters	
NT-proBNP (pg/mL)	**1.000028 (1.000015–1.000041)**
Creatinine (mg/dL)	**1.11 (1.04–1.19)**
Neutrophil count (n/microl)	**1.10 (1.07–1.13)**
eGFR (ml/min/1.73 m^2^)	**0.98 (0.97–0.99)**
CRP (mg/dL)	**1.07 (1.05–1.10)**
Systolic BP (mmHg)	0.99 (0.98–1.00)
Cluster (ref. 1)	
2	**1.96 (1.28–3.01)**
3	**2.87 (1.62–5.07)**

Bold indicates significance. Dyslipidemia, hypertension, chronic lung diseases, and diabetes mellitus were defined according to individual medical history and/or respective treatments taken at home. Atrial fibrillation, myocardial infarction, and stroke are referred to as previous events in the history of the patient. Laboratory parameters and blood pressure are expressed as continuous variables. HRs for diabetes mellitus, hypertension, atrial fibrillation, dementia, myocardial infarction, concurrent bacterial infection, stroke, and dyslipidemia refer to the presence of disease in reference to the absence of disease. NT-proBNP: N-terminal pro-B-type natriuretic peptide; eGFR: estimated glomerular filtration rate; CRP: C-reactive protein; BP: blood pressure.

**Table 3:** Accuracy of clusters and net reclassification improvement for death during hospitalization.

Outcome	Addition	AUC (95% CI)	Overall NRI (95%CI)	ΔAUC (95%CI)	*p*
Death (*n* = 138)		0.60 (0.54–0.66)			
	CFS		0.57 (0.24–0.76)	0.12 (0.07–0.17)	<0.001

## Data Availability

The data presented in this study are available on request from the corresponding author.
